# Deformation Behavior and Fracture Patterns of Laminated PEEK- and PI-Based Composites with Various Carbon-Fiber Reinforcement

**DOI:** 10.3390/polym13142268

**Published:** 2021-07-10

**Authors:** Pavel V Kosmachev, Vladislav O Alexenko, Svetlana A Bochkareva, Sergey V Panin

**Affiliations:** 1 Institute of Strength Physics and Materials Science SB RAS, 2/4, Akademicheskii Pr., 634055 Tomsk, Russia; vl.aleksenko@mail.ru (V.O.A.); svetlanab7@yandex.ru (S.A.B.); svp@ispms.ru (S.V.P.); 2 Department of Materials Science, Engineering School of Advanced Manufacturing Technologies, National Research Tomsk Polytechnic University, 30, Lenina Pr., 634050 Tomsk, Russia

**Keywords:** carbon fiber, PEEK, PI, laminates, adhesion, stress-strain state, FEM

## Abstract

Laminated composites based on polyetheretherketone (PEEK) and polyimide (PI) matrices were fabricated by hot compression. Reinforcing materials (unidirectional carbon-fiber (CF) tapes or carbon fabric) and their layout patterns were varied. Stress–strain diagrams after three-point flexural tests were analyzed, and both lateral faces of the fractured specimens and fractured surfaces (obtained by optical and scanning electron microscopy, respectively) were studied. It was shown that the laminated composites possessed the maximum mechanical properties (flexural elastic modulus and strength) in the case of the unidirectional CF (0°/0°) layout. These composites were also not subjected to catastrophic failure during the tests. The PEEK-based composites showed twice the flexural strength of the PI-based ones (0.4 and 0.2 GPa, respectively), while the flexural modulus was four times higher (60 and 15 GPa, correspondently). The reason was associated with different melt flowability of the used polymer matrices and varied inter- (intra)layer adhesion levels. The effect of adhesion was additionally studied by computer simulation using a developed two-dimensional FE-model. It considered initial defects between the binder and CF, as well as subsequent delamination and failure under loads. Based on the developed FE-model, the influence of defects and delamination on the strength properties of the composites was shown at different stress states, and the corresponding quantitative estimates were reported. Moreover, another model was developed to determine the three-point flexural properties of the composites reinforced with CF and carbon fabric, taking into account different fiber layouts. It was shown within this model framework that the flexural strength of the studied composites could be increased by an order of magnitude by enhancing the adhesion level (considered through the contact area between CF and the binder).

## 1. Introduction

The design of structural composite materials based on thermoplastic polymers reinforced with carbon fibers (CF) and fabrics is a major trend in advanced manufacturing around the world. For example, this topic is most relevant for the aerospace industry, where materials with high specific strength levels (the strength to density ratio) are required, including at elevated operating temperatures. Currently, research and development are being carried out in the field of the fabrication of laminated polymer composites reinforced with continuous CF. This enables the making of products with greatly improved mechanical properties [1,2,3,4]. Typically, the laminated composites are produced on the base of epoxy- or carbon-based polymers. However, the use of high-temperature high-strength thermoplastic matrices enables to solve a number of urgent issues, for example, to exclude their brittleness. In addition, this opens up the prospect of manufacturing parts from pre-impregnated ones using conventional industrial equipment. Polyetheretherketone (PEEK), polyimide (PI), polyphenylene sulfide (PPS), and a number of others should be mentioned as high-temperature thermoplastic materials recommended for the fabrication of laminated reinforced composites [5]. The procedures required for manufacturing parts from laminated composites reinforced with continuous CF include the layer-by-layer layout of polymer powders or films, and CF tapes or fabric. For instance, the paper [6] describes the properties of the laminated composite consisting of nine layers of the PEEK film 300 μm thick (1000-300G, Victrex) and eight CF layers (3K-T300, Toray Industries). The layout pattern was characterized by the (0°/90°) scheme. The composite possessed the following properties: a flexural tensile strength of 400 MPa and an elastic modulus of 20 GPa, as well as an interlaminar shear strength of 50 MPa. Moreover, it was shown in [7,8,9] that the highest mechanical properties of the PI-based composites had been achieved with the continuous CF reinforcement.

Directional reinforcement with continuous CF is a well-known method for enhancing the strength properties of the laminated composites. In this case, the layout directions can vary from layer to layer, which also affects their fracture behavior [6]. The mechanical characteristics of the composites after various modes of weaving and the layout of carbon fabric were reported in some papers. It is shown in [7] that the mechanical properties of the transversely-reinforced one (0°/90°/45°/–45°) are 30–40% lower in comparison with those for the unidirectional scheme (0°/0°). However, it was noted in [8] that the anisotropy of the properties of the composites reinforced with CF manifested due to an unidirectional weaving and layout, while it possessed isotropic properties after using the (0°/90°/45°/–45°) scheme. Despite all the advantages of the laminated polymer composites, one of the key challenges is weak adhesion at the CF/matrix interface due to different polarities and the absence of reactive functional groups in the polymer, as well as smooth CF surfaces. The possible delamination of the composites severely limits their applications. Various methods of CF surface modification are being developed to solve this issue. Plasma treatment [10,11,12] results in the active formation of functional groups, an increase in the surface energy, and the development of rougher CF surfaces, which enhances the polymer-to-CF mechanical adhesion. During CF thermal oxidation [13,14], the weakly-bound defective carbon surface layer is removed, oxygen chemisorption occurs at the edge carbon atoms, and oxygen-containing functional groups are formed that can react with the polymer matrix. Thus, an increase in the adhesive interaction at the interphase boundaries is ensured. Depending on the processing conditions, the structure and surface morphology of the modified CF can vary significantly.

One of the approaches for improving the interfacial adhesion of the laminated polymer composites is to load them with nano-fillers. Due to their high specific surface area, which promotes the formation of more interphase boundaries and a better interaction between the filler and the matrix, they have been widely used for the modification of laminated composites in recent years. It has been shown in [15,16] that loading the polymer matrix with nano-silica, halloysite, and fullerene-like tungsten disulfide improves its damping properties, fatigue life, and impact toughness. Another modification method is the deposition of the nano-filler coating on the CF surfaces [17]. Using coupling agents, which have good compatibility with polymers [9,18,19], causes the formation of hydrogen bonds and π–π interactions between the coupling agent and the matrix. This significantly improves the interfacial adhesion of the composites. In addition to material science aspects, an effective way to improve the mechanical characteristics of the laminated polymer composites is to optimize their sintering parameters. It has been shown in [20,21,22], using PEEK as an example, that optimization of the molding temperature and pressure, holding time, and cooling rate enable enhancement of the elastic modulus, ultimate tensile, and interlayer shear strength. As a result of better wetting of CF by the polymer matrix, a decrease in active defects and discontinuities has been observed for the composites.

In these studies, high-strength laminated PEEK- and PI-based composites have been fabricated and additionally reinforced with continuous CF with various layouts. It should be noted that the role of the reinforcement behavior and adhesion between a binder and CF is detailed for laminated epoxy-based composites. However, high-strength thermoplastics (such as PEEK, PPS, PI, etc.) in the molten state interact with reinforcing fibers (fabrics) in a completely different way (in contrast to the epoxy binder, which is characterized by high flowability and low plasticity). The deformation behavior of such composites under mechanical loading (in particular, bending) is also different from the epoxy-based ones.

Despite comparable strength properties (the elastic modulus and ultimate tensile strength), PEEK- and PI-based composites differ in the following characteristics: (i) melt flow index (MFI); (ii) chemical nature/activity; (iii) plasticity, etc. As a result, both formed structures and deformation behavior are varied. Therefore, a comparative study of laminated PEEK- and PI-based composites fabricated under identical conditions seems relevant.

On the other hand, the decisive effect of interfacial and interlayer adhesion on the deformation and strength properties of laminated composites is known. Since it has not been possible to vary the aspect of adhesion in the experimental investigations, such studies have been carried out within the framework of the theoretical part of the research. To assess the influence of adhesion on the mechanical properties of the composites and understand the possibilities of their improvement, a finite element model has been developed, taking into account the presence of initial interfacial discontinuities (defects), different levels of interfacial bonding, as well as the delamination and cohesive fracture processes.

To analyze the complex deformation and fracture mechanisms for the laminated composites and identify the key factors that have a significant effect on the mechanical properties of products, it is necessary to develop a correct model. For this purpose, computer simulation methods are often applied, including numerical ones implemented in various commercial software packages. For example, the finite element method (FEM) enables prediction of a wide range of properties and phenomena, such as the CF/matrix interfacial adhesion, crack initiation and propagation, strength, viscoelastic and plastic characteristics, as well as the ability to absorb mechanical energy.

Many authors distinguish constitutive and structural FEM-based models [23]. For example, Y. Chen et al. [24] have developed one for linear viscoelastic CF-reinforced composites using a time-domain homogenization procedure. Additionally, L.B. Andraju and G. Raju [25] have suggested a progressive damage model implemented in the Abaqus FEM package, which includes 3D Hashin fracture criteria, as well as cohesive zone templates to study intra- and interlaminar damages in composites reinforced using unidirectional (0)20, diagonally (0/90)5s, and different-angle (45/0/–45/90/0)2s schemes. M.A. Pérez et al. [26] have predicted the composite characteristics by the matrix-enhanced mixing theory, which is a simplified version of the theory of sequential and parallel mixing. The model does not require the use of an iterative procedure, as well as the tangential stiffness tensor calculation. B.M. Luccioni [27] has developed a general constitutive one for CF-reinforced laminated composites that is a generalization of the classical mixture theory, which considers the stress–strain relationship for the composite components and the main material symmetry directions. Recently, multilevel approaches for the analysis of laminated composites have been widely implemented. For instance, J. Lorca et al. [28] have presented their research results in a multiscale simulation of the CF-reinforced polymer composites, which was performed by calculating the properties of one ‘sub-object’ (for example, a separate layer) in its dimensional scale, then homogenizing the results within the constitutive model and ‘transferring’ these data to the next scale level to determine the mechanical behavior of the entire object (for instance, a laminate composite). X.Q. Peng and J. Cao [29] have presented a non-orthogonal constitutive model for calculating the characteristics of the anisotropic behavior of carbon-fabric reinforced composites under severe loads.

Some models of laminated composites, which take into account CF layouts, have been described by many authors to date. Nevertheless, all the ones developed considered the presence of initial defects, and the fracture process dynamics remains relevant. In this case, it is necessary to consider the complex effect of possible failure mechanisms for the composites under different stress states. It should be noted that there are no generally accepted models and methods in this direction yet.

The aim of these studies has been to analyze the deformation behavior of the laminated PEEK- and PI-based composites reinforced with continuous CF. Within the framework of the experimental and theoretical investigations, the CF layouts have been varied. The effect of interlayer adhesion on the mechanical response of the composites has been assessed by computer simulation of the process using a developed finite element model.

## 2. Materials and Methods

A PEEK film 250 μm thick ‘Aptiv 2000’ (Victrex Plc, Thorton-Cleveleys, UK), PEEK powder ‘PEEK 450 PF’ (Victrex Plc, Thorton-Cleveleys, UK), with a particle size of 50 μm, and a PI powder 1600 (Solver Polyimide, Jiande, China) with a particle size of 16 μm were used to fabricate the polymer matrices. A unidirectional CF tape ‘12K-300-230’ (UMATEX, Moscow, Russia) with a surface density of 230 g/m^2^, a tensile modulus of 245 GPa, and a tensile strength of more than 4.9 GPa, as well as the 0/90-50K-1270-106 biaxial carbon fabric with a surface density of 106 g/m^2^, a tensile modulus of 420 GPa (HC COMPOSIT, Moscow, Russia) were loaded for the reinforcement purpose. The thicknesses of the carbon tapes and the biaxial fabrics were 250 and 110 µm, respectively.

The MFI of the polymers was determined using a plunger laboratory extruder-UE-MSL (UGN lab., in compliance with ASTM D 1238) under the following conditions: *T* = 370°(PI); 400 °C (PEEK), load P = 100 N.

Table 1 presents the MFI values for the PEEK-powder, PEEK-film, and PI-powder samples. Its levels were comparable for the PEEK-powder and PEEK-film ones, while the PI-powder sample was characterized by almost half their melt flow rate.

**Figure 1:**
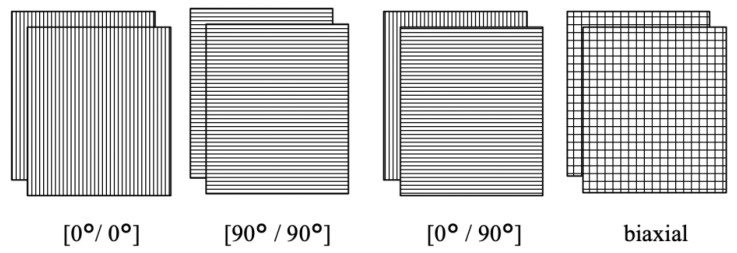
Schemes of the CF layout patterns (the polymer powder located between them).

The CF/matrix ratio was 40/60 vol.%, which has been determined on the basis of the published data analysis, as it is the most effective for improving the mechanical characteristics. The composites were reinforced with continuous CF using various layout patterns such as (0°/0°), (90°/90°), (0°/90°), as well as layers of the biaxial (braided) fabric (Figure 1).

**Figure 2:**
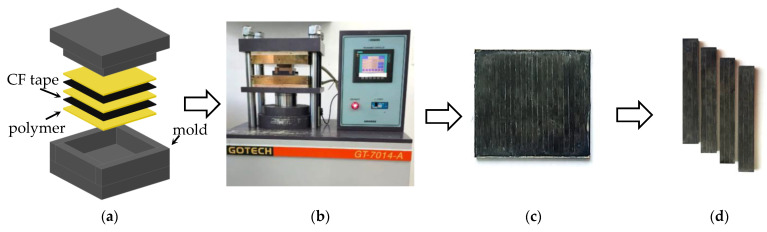
The step-by-step diagram of the process of fabricating laminated composite samples: stacking layers (**a**); hot compressing (**b**); plates (blanks) for cutting specimens for the mechanical tests (**c**); ready specimens (**d**).

After packing in a mold (Figure 2a), the composite samples were fabricated by hot compressing. A ‘GT-7014-A’ thermo-hydraulic press (GOTECH Testing Machines Inc., Taichug, Taiwan, Figure 2b) was used at a specific pressure of 10 MPa and a temperature of 400 °C. The cooling rate was 2 °C/min. After hot compressing, the 70 × 65 × 3.5 mm composite plates (blanks) were obtained (Figure 2c), from which specimens for mechanical tests were cut.

The mechanical properties of the composites were determined by three-point flexural tests using an ‘Instron 5582’ electromechanical testing machine in accordance with ASTM D790-10. The specimens for the mechanical tests were 70 × 10 × 3.5 mm in size (Figure 2d).

A ‘Neophot 2’ optical microscope (Carl Zeiss, Jena, Germany) was used to study the cross-sectional surfaces of the fractured specimens after the three-point flexural tests. Structural analysis of the composites was carried out using a ‘Quanta 200 3D’ scanning electron microscope (SEM) with an electron (focused) beam (FEI Company, Hillsboro, OR, USA) at an accelerating voltage of 20 kV. A thin copper film had been preliminarily deposited on the fracture surfaces to ensure the electrical conductivity of the composites.

## 3. Results and Discussion

### 3.1. Experimental Part

#### 3.1.1. Comparison between Powder and Film PEEK-Based Composites

Manufacturers supplied PEEK as a powder or a film. To compare the effect of the PEEK feedstock type, the laminated PEEK-based composites were made from both. The thickness of the polymer layers was identical in both cases. Obviously, the use of the film compared to the powder enabled better reproducibility of the polymer layer thicknesses. Table 2 shows the mechanical properties of both PEEK-powder and PEEK-film composites, which were rather similar. According to the authors, such an effect on the mechanical properties of composites was due to matching these parameters for both PEEK feedstock types, as well as the identity of the formed structure. However, the use of the film was more convenient in terms of technology. Therefore, only the PEEK film was loaded further.

However, for further comparison, the PEEK- and PI-based composites had been fabricated in a slightly different way, which could affect the formed structures. In the case of PEEK, the film and CF tapes (or the carbon fabrics) were used. In another case, the authors had been unable to purchase a ready-made PI film. For this reason, much like in the case of PEEK, a volume of the powder identical in weight had been poured between the reinforcing CF layers. Respectively, PI sintering had taken place during the formation of the entire laminated composite.

#### 3.1.2. Three-Point Bending Flexural Test

**Figure 3:**
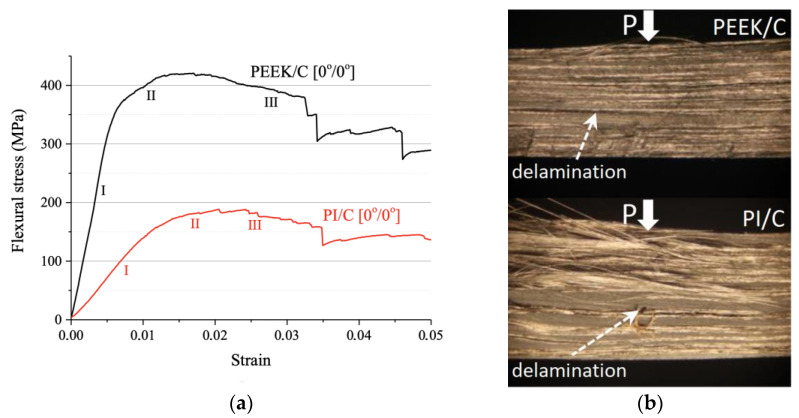
The flexural stress–strain curves (**a**) and optical images of the specimen cross-sections after the three-point flexural tests (**b**) for the PEEK/CF (0°/0°) and PI/CF (0°/0°) composites.

Figure 3a shows flexural stress–strain curves for both PEEK/CF (0°/0°) and PI/CF (0°/0°) composites. At the linear elasticity stage (I), a higher angle of inclination was characteristic of the PEEK/CF (0°/0°) one. The calculated elastic modulus values for the PEEK/CF (0°/0°) and PI/CF (0°/0°) composites were 59.5 ± 3.7 and 15.0 ± 2.0 GPa, respectively. It should be noted that the flexural moduli differ to a much lesser extent for the matrices (4.2 and 2.4 GPa), while the elastic moduli were quite close in magnitude (2.8 and 2.6 GPa). This was followed by the inelastic strain stage (II), which was characterized by achieving the maximum stresses. For the PEEK/CF (0°/0°) composite, its value was 422.8 ± 12.5 MPa at a strain of 0.017, while the peak stress was 190.2 ± 18.7 MPa at a strain of 0.020 for the PI/CF (0°/0°) one. Overcoming the maximum stress point resulted in the development of the fracture process, which corresponded to stage (III) of the analyzed diagrams. In this case, the formation of cracks occurred mainly along the layer boundaries according to the delamination mechanism. In the case of the PI/CF (0°/0°) composite, delamination was strongly pronounced in the reinforcing layers (Figure 3b), while the PEEK/CF (0°/0°) composite resisted local delamination better (apparently due to higher interlayer adhesion, discussed below).

Stage (III) was the most extended. However, macro-fracture of the composite did not occur despite the formation of pronounced stress-drop sections that corresponded to the formation or propagation of cracks. After reaching a strain of 0.050, the mechanical test was stopped. Thus, both composites appeared to be resistant to degradation, which could be classified as ‘damage tolerance’. Therefore, the revealed difference in strength properties of the laminated PEEK- and PI-based composites was due not only to the variations in the corresponding characteristics of the polymer binders. This was confirmed by the results of the tests of such composites with the transverse layout (reported below).

**Figure 4:**
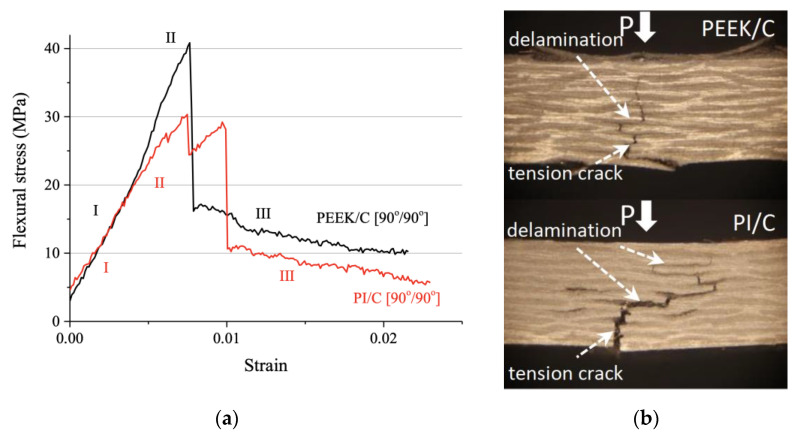
The flexural stress–strain curves (**a**) and optical images of the specimen cross-sections after the three-point flexural tests (**b**) for the PEEK/CF (90°/90°) and PI/CF (90°/90°) composites.

Figure 4a presents the flexural stress–strain curves for the PEEK/CF (90°/90°) and PI/CF (90°/90°) composites. At the linear elasticity stage (I), the diagrams of both composites almost coincided. The elastic modulus values were also factually equal for the PEEK/CF (90°/90°) and PI/CF (90°/90°) much like for the case of the PEEK ones (4.1 ± 0.3 and 4.1 ± 0.5 GPa, respectively). The inelastic strain stage (II) was accompanied by an increase in strain hardening for the PEEK/CF (90°/90°) composite, while a slight softening was observed for the PI/CF (90°/90°) one. The end of stage (II) corresponded to the peak stress and the first (transverse) crack initiation. As a result, stresses dropped sharply, which actually corresponded to the loss of bearing capacity at the beginning of stage (III). At the maximum load, stresses were about 42.3 ± 6.4 MPa for the PEEK/CF (90°/90°) composite and 30.1 ± 4.1 MPa for the PI/CF (90°/90°) one with identical strains of 0.008. For the PI/CF (90°/90°) composite, two falling regions were at stage (III), according to Figure 4a. This was because of the main transverse crack deceleration due to its propagation along the layer boundaries. Thus, the fracture of the transversely-reinforced laminated composites was associated with the development of main transverse cracks initiated from the lower edge of the specimen under tensile stresses. These cracks could be inhibited when they reach the interlayer boundaries.

**Figure 5:**
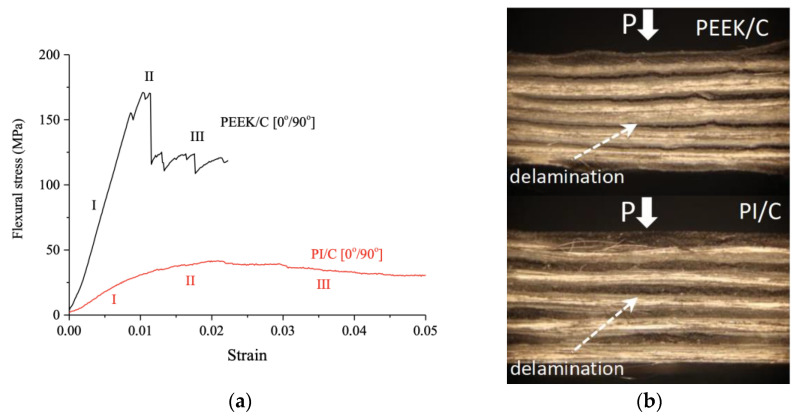
The flexural stress–strain curves (**a**) and optical images of the specimen cross-sections after the three-point flexural tests (**b**) for the PEEK/CF (0°/90°) and PI/CF (0°/90°) composites.

Figure 5a shows the flexural stress–strain curves for the PEEK/CF (0°/90°) and PI/CF (0°/90°) composites. In this case, variations of the strength properties of the two types of laminated composites reached the maximum level. First of all, a higher angle of inclination was again characteristic of the PEEK/CF (0°/90°) composite at the linear elasticity stage (I). The elastic modulus values were 19.8 ± 7.2 and 3.8 ± 1.1 GPa for the PEEK/CF (0°/90°) and PI/CF (0°/90°) composites, respectively. For the PEEK/CF (0°/90°) one, this was significantly higher than for the transverse CF reinforcement case, while the values were quite close for the PI/CF (0°/90°) composite. Moreover, stage (II) varied markedly for these polymer matrices. Thus, reaching the highest strain was accompanied by a sharp drop in stress level for the PEEK/CF (0°/90°) one. However, this could not be called a loss of bearing capacity since the composite continued to resist deformation at an almost constant load–Stage (III). At its maximum level, the stress was 173.8 ± 26.8 MPa at a strain of 0.010. The PEEK/CF (0°/90°) composite was characterized by multiple local strain drop zones at stage (III). This was associated with the development of the delamination process (Figure 5b). The PEEK/CF (0°/90°) composite fractured at $\varepsilon_{B}^{U}$ = 0.023. For the PI/CF (0°/90°) one, boundaries between the stages were blurred due to low strength properties. However, there was no pronounced stress drop in this case (Figure 5a). The maximum stress was 43.0 ± 5.2 MPa, which corresponded to a strain of 0.020. Thus, the main deformation macro-mechanism was the longitudinal delamination for both PEEK- and PI-based composites with a (0°/90°) CF layout pattern.

**Figure 6:**
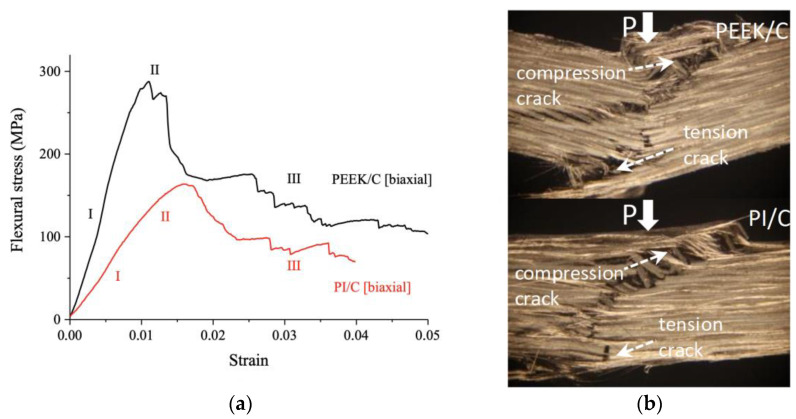
The flexural stress–strain curves (**a**) and optical images of the specimen cross-sections after the three-point flexural tests (**b**) for the PEEK/CF (biaxial) and PI/CF (biaxial) composites.

Figure 6a presents the flexural stress–strain curves for the PEEK/CF (biaxial) and PI/CF (biaxial) composites. In this case, the strength properties were lower than those for the unidirectionally reinforced ones but higher than in the transverse and alternate CF layout cases. At the linear elasticity stage (I), the PEEK/CF (biaxial) composite, as expected, possessed a higher slope angle. The elasticity modulus levels were 27.4 ± 0.3 and 10.8 ± 1.1 GPa for the PEEK/CF (biaxial) and PI/CF (biaxial) composites, respectively. For the PEEK/CF (biaxial) one, the maximum stress was 277.2 ± 34.9 MPa at a strain of 0.011. For the PI/CF (biaxial) composite, the maximum stress was 158.4 ± 1.2 MPa at a strain of 0.016. This was followed by the stress drop stage (III). However, it was impossible to draw conclusions about a complete loss of bearing capacity (like in the case of alternate CF layouts, considered above). Compared to other layout patterns, biaxial carbon fabric composites were less prone to delamination (Figure 6b). The main crack was oriented at an angle to the loading axis, and a series of local delaminations were observed. In this case, the crack was retarded at each next interlayer boundary. Frequently, two main cracks could be distinguished, which had propagated from each of the composite flat faces (Figure 6b). Thus, the composites reinforced with biaxial fabrics did not show maximum strength characteristics, but their fracture type could be classified as damage tolerant. In addition, they are characterized by anisotropy of the properties, which was extremely important when the predominant load direction could not be distinguished. It should also be noted that the PEEK/CF (biaxial) composite did not fracture up to $\varepsilon_{B}^{U}=0.05$, while the PI/CF (biaxial) one failed at $\varepsilon_{B}^{U}=0.04$. Most likely, the reason was associated with the difference between interfacial and interlayer adhesion.

The conducted analysis of the flexural stress–strain curves showed that fracture of the studied laminated composites was a discrete-continuous process, representing a set of separate (particular) acts. According to published data, discontinuities occurred first at the CF/matrix interfaces upon loading. Further, the following processes developed: fracture of individual CF, interlayer delamination, decay of polymer layers, and, finally, failure of the composite [30]. In any case, increasing interfacial (interlayer) adhesion was crucial for improving strength properties. In these studies, this fact manifested itself in significantly lower mechanical characteristics of the PI-based composites, which, according to the authors, was associated with a lower PI flowability and its adhesion with reinforcing CF.

**Figure 7:**
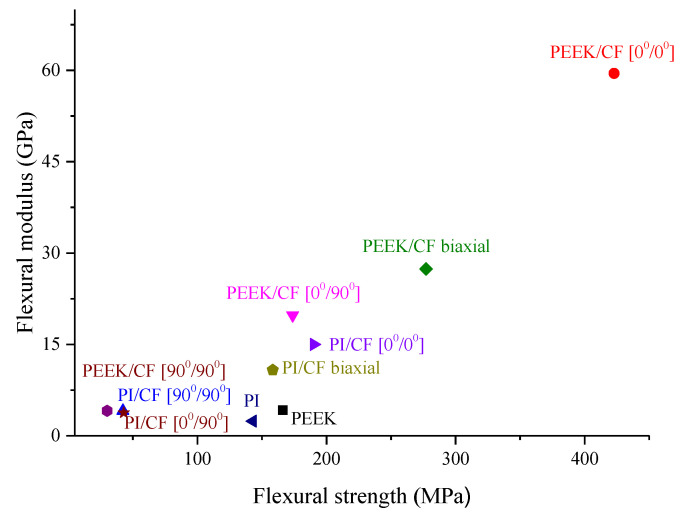
The summarized mechanical test results for both PEEK- and PI-based composites in the flexural ‘strength–modulus’ coordinates.

The physical and mechanical properties of the studied composites are summarized in Table 3. According to the obtained results, the maximum increase in the strength characteristics (both flexural modulus and strength) was achieved for the unidirectional composites with the (0°/0°) layout pattern. In addition, the graphical systematization of the data is shown in Figure 7 in the ‘modulus–strength’ coordinates.

Thus, the PEEK/CF (0°/0°) composite possessed the maximum strength properties when the load and CF axes coincided; the fabric PEEK/CF (biaxial) one then followed. The PI-based composites were noticeably less durable than the PEEK-based ones. From the point of view of the effect of the layout, the obtained data were consistent with all the canons of the composite mechanics. However, the different influence of the polymer binder type did not follow from the variations of their strength properties.

#### 3.1.3. Structural Studies

**Figure 8:**
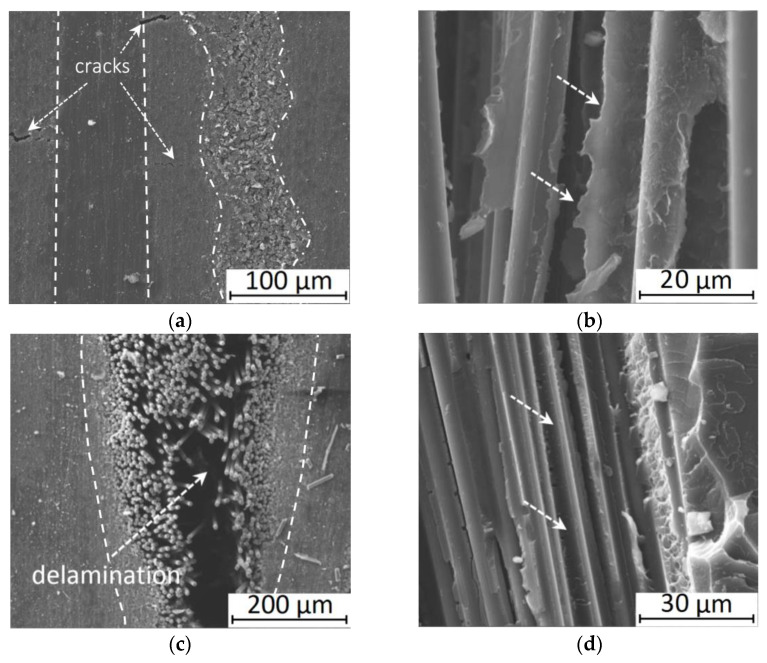
SEM micrographs of the composite structures. Interlayer level: PEEK/CF (**a**), PI/CF (**c**). Interfacial level: PEEK/CF (**b**), PI/CF (**d**).

The analysis of the reasons for such characteristics, which were lower than the results of simulation and experimental data obtained by other authors [6,7,8,9], was carried out using SEM micrographs of the composite structures (Figure 8). The interlayer boundary between the PEEK film and CF (shown by the dashed line) was straight and did not contain any discontinuities. Moreover, the molten polymer had effectively impregnated the CF layer to form a wavy interface (shown by the dash-dotted line). A similar pattern was observed from the side of the neighboring PEEK layer. However, the area between them turned out to be worse impregnated binder material. At the same time, microcracks were found in the transitional (well-impregnated) zone between the mating layers (shown by arrows in Figure 8a).

Therefore, the reinforcing layer adhered rather tightly to the polymer matrix in the PEEK-based composites. There were small regions with insufficient adhesion and even microcracks, which, however, were not catastrophic in terms of the macro-properties.

On the other hand, pronounced delamination up to several tens of micrometers width was observed inside the CF layer (tape) of the PI-based composite (Figure 8c). However, much like the PEEK case at the CF/PI interface (shown in dotted line), satisfactory adhesion was found due to the interaction between the molten PI powder and CF upon hot compressing. However, the well-impregnated transition layer inside the PI film did not appear. According to the authors, the almost two times lower MFI level for PI did not enable the CF-tape layer to be sufficiently saturated, which caused similar longitudinal delamination. This assumption was consistent with the absence of visible microcracks in the transition layer between the polymer and the CF tape. Therefore, the formation of such (local) delamination was the main reason for the decrease in the strength properties of the laminated PI-based composite during the three-point bending test.

The results presented in Figure 8a,c characterized interlayer adhesion, while the data presented in Figure 8b,d reflected interfacial adhesion at the CF/matrix interface. The specimens, fractured upon the flexural tests, were analyzed. In the case of the PEEK-based composite, fragments of the polymer matrix were visible on the CF surface (Figure 8b). PEEK enveloped CF provided an acceptable level of interfacial adhesion. In the case of the PI-based composite (Figure 8d), fragments of the polymer matrix were also found on the CF surface. However, their number was less than in the case of the PEEK-based ones. PI polymer enveloped CF and possessed satisfactory adhesion to them.

Thus, the CF/polymer interfacial bond could be formed despite the presence of the technological coupling agent for epoxy polymers on the CF surface. According to the authors, its level was slighter higher for PEEK, but the difference was not significant. The revealed significant variation of the strength properties of the composites with polymers of both types was associated, first of all, with various levels of interlayer adhesion. Since it was not possible to purposefully vary the adhesion value in the experimental part, and the presence of defects in the transitional layers was rather random, parametric studies of the influence of such factors were carried out then. A convenient tool for this purpose was computer simulation using the finite element method.

### 3.2. Computational Part

#### 3.2.1. Problem Statement

In order to assess the mechanical properties of the laminated composites, models were proposed that considered the presence of discontinuities, realized interphase delamination, and fracture under loads. On their basis, the authors analyzed the effect of interfacial adhesion and imperfect contact between CF layers and the polymer binders on the mechanical properties of the composites. The models were implemented in the author’s software package based on the finite element method. The code was written in Fortran and compiled using Intel Parallel Studio XE Composter Editor for Fortran Windows.

**Figure 9:**
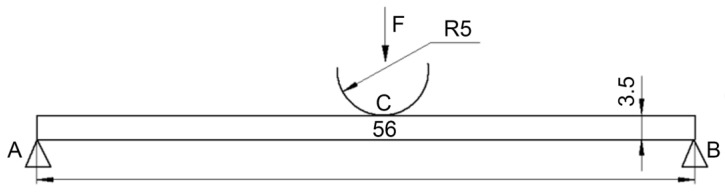
The computational domain diagram for simulation of the three-point flexural tests.

Since it was not possible to carry out parametric studies of the effect of interlayer adhesion on the strain and strength properties of the composites within the experimental section of the work, this issue was considered theoretically. Numerical simulation of the three-point flexural tests according to ISO 14125:1998 was carried out. The computational domain diagram is shown in Figure 9.

To determine the stress–strain state parameters of the samples, the problem of the plane stress state was solved. The calculations were carried out using the author’s software package mentioned above. Triangular simplex elements were used in the calculation.

**Figure 10:**
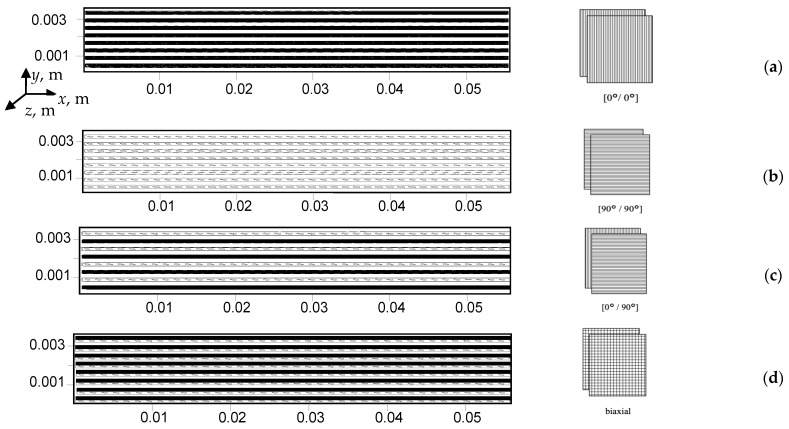
Computational schemes corresponding to different CF layouts: (**a**)—(0°/0°); (**b**)—(90°/90°); (**c**)—(0°/90°); (**d**)—(biaxial).

Figure 10 shows schematic diagrams of computational domains for the composites consisting of polymer layers alternating with polymer-impregnated CF ones (monolayers), simulating both unidirectional and bidirectional fabrics. The CF layers are shown as narrow, filled rectangles, the shading of which represents the longitudinal or transverse position of CF in the layer:

- Option 1 (Figure 10a): polymer layers alternate with CF layers directed along the *x*-axis;
- Option 2 (Figure 10b): along the *z*-axis;
- Option 3 (Figure 10c): a layer of polymer, a layer with CF directed along the *x*-axis, a layer of polymer, then a layer of fibers along the *z*-axis, then the sequence was repeated;
- Option 4 (Figure 10d): the polymer layers alternate with the layers of CF directed along the *x* and *z* axes, which imitated fabric, then the sequence was repeated.

In the layers, CF was represented as bundles with an average diameter of 180 μm; the layer thickness was assumed to be the same (hereinafter, CF means a bundle of fibers). The number of CF monolayers in the specimens was 11. The layers were distributed so that the lower and upper ones corresponded to CF. The CF strength was 4.9 GPa, and their elasticity modulus was 240 GPa.

#### 3.2.2. Calculation of the Mechanical Properties of one Impregnated CF Layer (Monolayer)

**Figure 11:**
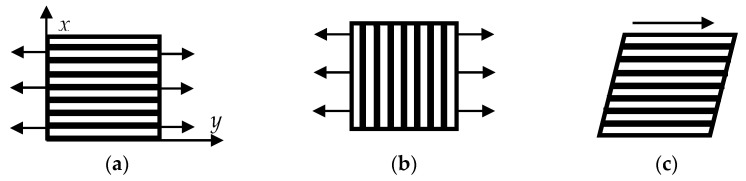
The schemes of composite-impregnated CF layers under tension: (**a**) along CF; (**b**) across CF; (**c**) shear along CF.

The calculation of the mechanical properties of one composite-impregnated CF layer (monolayer) was carried out on the basis of a flat SSS computational domain under tension along (Figure 11a) and across CF (Figure 11b), as well as shear along CF (Figure 11c).

**Figure 12:**
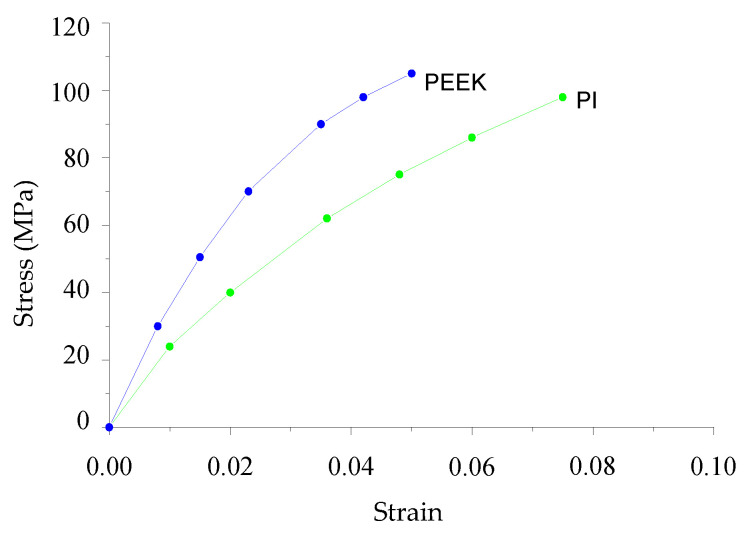
The stress–strain diagrams for PEEK and PI.

According to the first part of the paper, the CF content in one direction was 80 vol.%. Therefore, the CF/polymer ratio was taken 20/80 vol.% in one layer for the unidirectional fabric, but 40/60 vol.% for the biaxial one, since its surface density was 2.2 times less than in the first case. Thus, the free space between CF was 20% in a monolayer, and 40% in a fabric one. This space could be filled with polymer, which was taken into account when calculating the properties of one layer. The computational domain size was 3.5 × 3.5 mm. The non-linearity of the PEEK and PI tensile diagrams was also considered (Figure 12). For CF, the elastic strain condition was preset.

**Figure 13:**
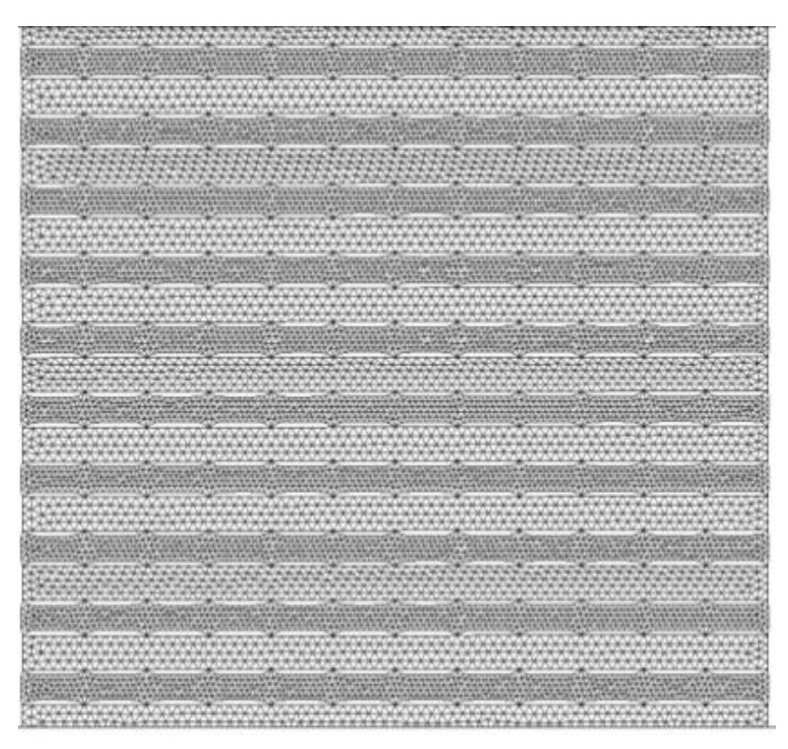
The computational domain with FEM mesh under tension across CF without any node discontinuities.

CF and the polymers were modeled as autonomous regions. The contact between them was considered by their connection in contacting nodes. The number of these nodes and the level of delamination stresses between them reflected the presence of discontinuities, i.e., when the materials contact partially in several spots and not over the entire surface area. The contacting nodes were distributed evenly along the CF length without any node discontinuities (Figure 13). For clarity, the extended FEM mesh is shown precisely for this case. To ensure bonding between CF and the matrix, the conditions for equality of displacements were in the contacting nodes, and corresponding changes were considered for the matrix stiffness [30].

The finite simplex-elements of a triangular shape were used, so if nodes *m* and *l* of one CF contacting triangle’s surface were taken as the main ones, and node *k* of another triangle’s contacting surface was considered dependent, then the displacement would be calculated through the movement of the main nodes:

*v_k_* = *v_l_*(1 − *h*) + *hv_m_*, (1)

*u_k_* = *u_l_*(1 − *h*) + *hu_m_*, (2)

where *u* and *v* are the displacement along the *x* and *y* axes, respectively; 0 ≤ *h* ≤ 1 defined the normalized distance of point *k* from node *l*:

(3) $$h=\sqrt{\frac{{\left(y_{k}-y_{l}\right)}^{2}+{\left(x_{k}-x_{l}\right)}^{2}}{{\left(y_{m}-y_{l}\right)}^{2}+{\left(x_{m}-x_{l}\right)}^{2}}}$$

Under tensile load along fiber orientation the following boundary conditions were established: displacements along the *x*-axis (*u*) were set to the right and left of the computational domain boundaries (Figure 10a). In this case, displacements along the *y*-axis (*u*) were equal to zero at these boundaries, while the lower and upper ones remained unoccupied, considering the imperfect contact between CF and the matrix (the materials were partially contacted in several spots, rather than over the entire surface area). The tangential (*τ*) and normal (*σ_n_*) stresses at the boundaries were equal to zero:

(4) $$\tau =0;\;\sigma_{n}=0$$

Under tensile load along CF, the conditions were additionally set for equality of displacements along the *y*-axis at intermediate points among the main contact nodes before delamination between the polymer and CF. This was done in order to avoid their intersection in the FEM mesh during compression in the transverse direction due to the Poisson effect. The boundary conditions were similar to those described above. After delamination, only the conditions of node displacements along the *y*-axis were used. Since CF moved in the opposite direction along the *y*-axis and pressed on the exfoliated polymer, these conditions were established on the lower CF edge in the lower half of the computational domain and on the upper polymer edge in its upper half.

Under tensile load and across CF (Figure 11b), displacements along the *y*-axis (*v*) were set step-by-step at the upper and lower boundaries of the computational domain (Figure 11a). Movements along the *x*-axis were zero at these boundaries, and the right and left ones were unoccupied.

For the case of tension across CF (Figure 10b), the following boundary conditions were preset: at the upper and lower boundaries of the computational domain (Figure 10a), displacements along the *y* (*v*) axis were assigned; displacements along the *x*-axis at these boundaries were equal to zero; normal and shear stresses were also equal to zero on the right and left boundaries. As for the tension along CF, the conditions of equality of displacements were set in the main contacting nodes (1, 2).

At each step, the polymer elastic modulus was corrected in every finite element (Figure 12), and the fracture criteria were checked, i.e., one of the stress tensor components reaching the limits equal to the yield point (the maximum stress criterion). The maximum strain criterion was also checked (the strain intensities corresponding to the yield point were taken as the limiting ones). If any criterion was met in an element, then its elastic modulus decreased by 100 times relative to the polymer level, and stresses were equal to zero in it. This corresponded to the element fracture. If the element in which the criterion was fulfilled contained a contact node, then delamination between CF and the polymer occurred (i.e., the corresponding mesh nodes were no longer in contact). The beginning of the limiting state (failure) of the composite as a whole was determined based on the fulfillment of one of two conditions. The first condition was met if the number of fractured elements reached 10% of the area of the corresponding material (typically, the polymer). The second was, if the number of delaminated nodes in one layer reached 50% of the amount of ones in this layer, then the composite was considered fractured.

**Figure 14:**
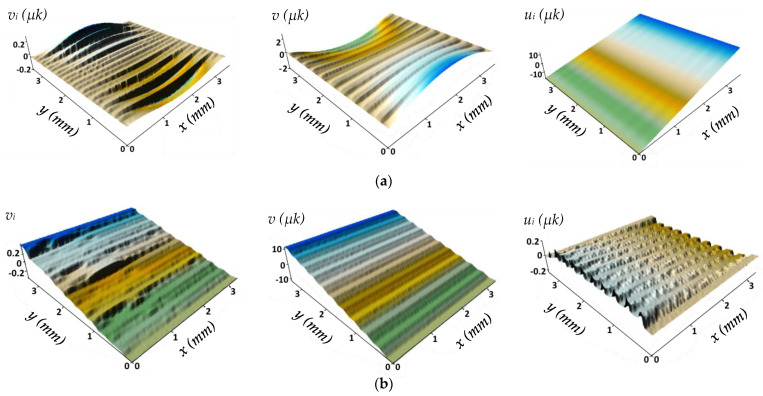
The computational domain with FEM mesh and displacement surfaces under tension: (**a**) along CF; (**b**) across CF with the 60/40 vol.% fiber-polymer ratio (*v_i_*—along the *y*-axis at one step and *v*—total, *u*—along the *x*-axis).

Figure 14 shows the surfaces of the displacements along (a) and across (b) CF Under the tensile load when the fracture criteria were fulfilled. In Figure 14a, delamination occurred along the surface of the displacements for one step (*v_i_*), and the movement direction changed to the opposite side, i.e., became unclenched. In the final stage, the composite fracture was not immediately reflected, but the transverse compression due to the Poisson effect occurred before delamination. Along the *x*-axis (*u*), the composite fracture was almost discreet since the displacements were insignificant. Delamination started at the extreme (upper and lower) CF both at the beginning and at the end, which had delaminated first (Figure 14a).

For one step of displacements along the *y*-axis (*v_i_*), partial polymer degradation and delamination at different locations could be observed in Figure 14b, which was reflected in the uneven movement nature. On the final surfaces for displacements along the *y* (*v_i_*) and *x* axes (*u*), the first fracture step was not visually reflected due to short-range displacements.

The tensile modulus was determined as the ratio of area-averaged stresses to strains. In the case of an analytical calculation at *E_F_* ≫ *E_M_*, the longitudinal composite elasticity modulus (in the reinforcement direction) was assessed by this parameter for CF and their relative content in the composites; the transverse one was found by the polymer elasticity modulus according to the following formulas [31]:

(5) $$E_{x}=E_{F}\cdot \varphi ,$$

(6) $$E_{y}=\frac{E_{m}\;}{1-\varphi }$$

Longitudinal tensile strength was calculated by the expression:

(7) $$\sigma_{X}=\sigma_{F}\cdot \varphi$$

For the CF/polymer ratio of 60/40 vol.%, the calculated longitudinal elasticity modulus was 144 GPa (148 GPa according to the numerical simulation), ultimate strength was 2940 GPa (2930 GPa according to the numerical simulation), and the transverse elasticity modulus for the PEEK-based composite was 9.38 GPa (10 GPa at the full contact (130 contacted points)). Therefore, the results of analytical and numerical calculations differed within 3%, which confirmed the correctness of the developed model and reflected the convergence of the obtained data. To achieve this, the finite element density was higher in the polymer region than in the CF area since the expected displacements were greater in this case.

**Figure 15:**
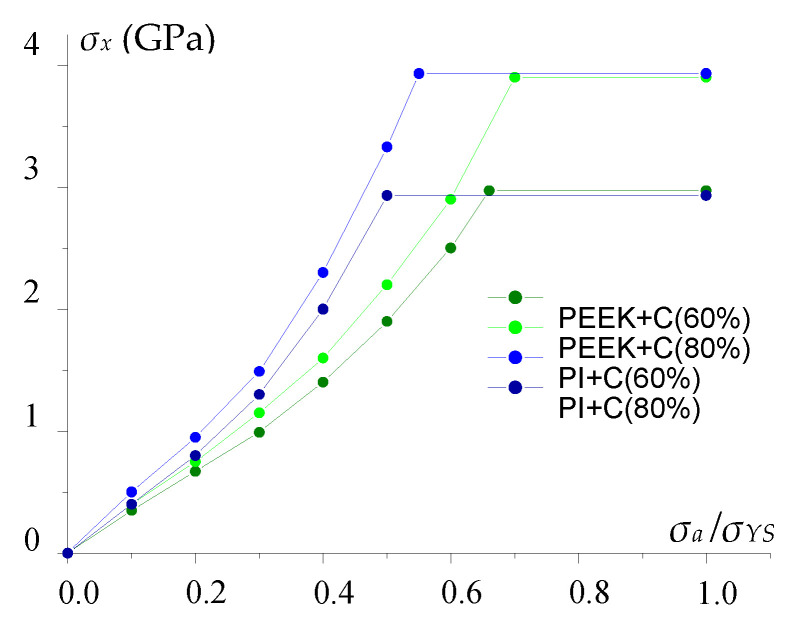
Change in tensile strength (*σ_x_*) under load along the *x*-axis (along CF) vs. peel stresses for the PEEK and PI-based composites with the 60/40 and 80/20 vol.% ratios.

Under tensile loading along CF, the polymer matrix deformed and fractured firstly and stronger CF only then (Figure 14). Therefore, imperfect contact (the number of contact points and the level of peel stresses) did not affect the elasticity modulus, but the CF/polymer ratio was decisive. However, the level of peel stresses affected the composite strength characteristics as a result of delamination (Figure 15) since the criterion for fracture was considered to be 50% delamination in one CF layer.

The maximum adhesion level was taken as tensile stresses (*σ_a_*) corresponding to the yield point (*σ_YS_*). Therefore, the change in adhesion level was determined by the *σ_a_*/*σ_YS_* ratio. At the same adhesion level, the strength properties were lower for the PEEK-based composites since stresses were higher, and failure occurred earlier in the same strains (Figure 11).

An increase in the number of contacting nodes caused the same results as in the calculation taking into account ideal adhesion. With the number of contact points above 130, the curves coincided. Its decreasing resulted in a reduction in tensile strength. This was determined by the fact that the specific load on the contact node was enhanced, and the criteria were met earlier in the vicinity of the contacting nodes. Consequently, the slope of the curves decreased. 

**Figure 16:**
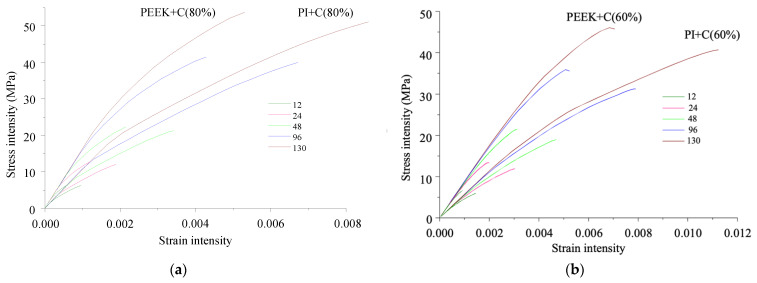
Stress vs. strain intensities of the PEEK- and PI-based composites under tension along the *y*-axis (across CF) with changing in the number of contacting points between the polymer and CF: the CF/polymer ratios of 80/20 (**a**) and 60/40 vol.% (**b**).

Figure 16 presents the stress intensity versus tensile strain rate dependencies for the PEEK- and PI-based composites along the *y*-axis (across CF) obtained at the adhesion level equal to the matrix yield point. With a decrease in the adhesion, the dependencies remained the same, but the limiting stresses were reduced proportionally to the decrease in the adhesion level. Under tensile load and across CF, fracture occurred as a result of the polymer delamination or fracture, depending on the adhesion level and the number of contacting nodes. With a decrease in the number of nodes and the adhesion level, only the delamination conditions were satisfied. The dependencies were nonlinear due to the same character of the matrix properties (Figure 12), as well as the delamination and fracture cases.

**Figure 17:**
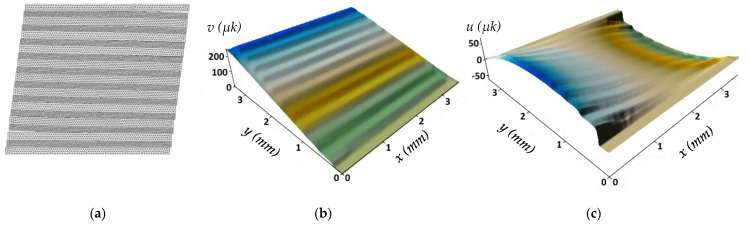
The computational domain of shear along CF for the CF/PEEK ratio of 60/40 vol.%: (**a**)—the FEM mesh; (**b**,**c**)—surfaces of displacement along the *y* and *x* axes, respectively.

The shear modulus along CF was determined according to the diagram in Figure 11c and was calculated as the ratio of total shear stresses to shear strains at the upper boundary of the computational domain. The following boundary conditions were assigned for shear along CF: displacements along the *y* (*v*) and *x* (*u*) axes were equal to zero at the lower boundary of the computational domain (Figure 10a); on the upper boundary, step-by-step displacements along the *x* (*u*) axis were set, but displacements along the *x* (*v*) axis were zero; normal and shear stresses were equal to zero on the right and left boundaries. The equality displacement conditions (1, 2) were assigned in the main contacting nodes, but condition (2) was in the intermediate ones. After delamination, condition (2) was only between layers with compressive stresses. The fracture occurred gradually according to both criteria. Delamination had begun at the lateral surfaces (Figure 17).

**Figure 18:**
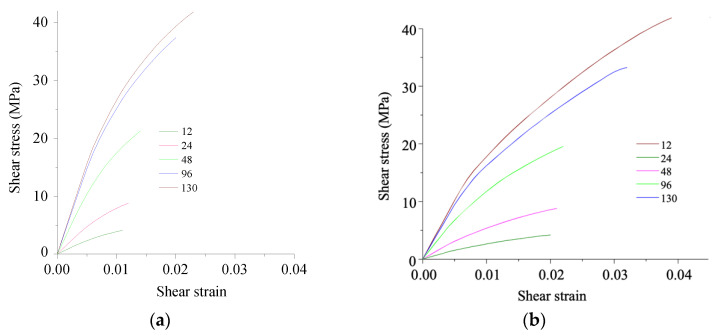
The shear stress–strain curves for the PEEK- (**a**) and PI-based (**b**) composites with changing contact points number.

Figure 18 shows shear stress–strain curves. The number of contact points affected both shear modulus and strength. As it decreased, the composite shear modulus reduced by about six times. Accordingly, the shear strength also decreased. The presented dependencies were obtained at an adhesion level equal to the matrix yield point. As it decreased, the limiting stresses reduced proportionally. In the flexural tests, shear strength significantly affected the result. Respectively, strength was determined by the adhesion level and the number of contact points.

The shear modulus was calculated using the formula:

(8) $$G=\frac{G_{m}G_{f}}{\varphi G_{m}+\left(1-\varphi \right)G_{f}},$$

where *G_m_* and *G_f_* are the matrices and CF shear modules; *φ* is the CF volumetric content.

At the CF/PEEK ratio of 60/40 vol.%, both results, obtained analytically and numerically for ideal adhesion (the number of contact points was 130 and more), were 3 GPa. This fact confirmed the correctness of the applied model.

The optimal CF/polymer ratio was (60–70)/(40–30) vol.% according to [31]. For the studied CF volume fraction of 60–80%, the mechanical properties of the composites could deteriorate due to the formation of large discontinuities between CF and the matrix. This was the so-called ‘CF overlapping effect’, due to which there was no matrix interlayer in the indicated areas. As a result, these matrix discontinuities reduced the strength characteristics and the elasticity modulus of the composites. In the applied model, discontinuities were determined by the number of contact points and significantly affected the composite properties, as shown above.

In the considered model, defects were determined by the number of contact points (Figure 15, Figure 16 and Figure 18), which significantly affected the strength properties of the composites at different stress states. Thus, the contact area (considering the number of contact points in the model) and strength decreased greatly with an increase in the defect quantity.

#### 3.2.3. Composite Characteristics in the Flexural Tests

Further, the experimentally determined properties of the composites were used to simulate their SSS in the flexural tests as the CF layer characteristics, excluding the shear modulus (Figure 10). In this case, delamination was taken into account only by the obtained properties of monolayers.

The CF/polymer ratio found in the experimental part was 40/60 vol.%. In computer simulation, the polymer filled 20% of the free space between unidirectional CF and 40% in one fabric layer. For this reason, the CF/polymer layer ratio was approximately 50/50 vol.%, while 60/40 vol.%. was in the fabric case, since 11 CF layers contained about 10% of the polymer (20% in the fabric case).

The following boundary conditions were set: displacements along the *y*-axis (*v*) were equal to zero at the corner points A and B from below, according to the specimen support condition.

The following boundary conditions were set: displacements along the *y*-axis (*v*) were equal to zero at the corner points A and B from below, according to the specimen support condition:

(9) $${v|}_{A}=0;\;{v|}_{B}=0$$

At point C, displacements along the *x*-axis (*u*) were zero, which enabled to maintain symmetry:

(10) $${u|}_{C}=0$$

At point C, a load was set step-by-step, distributed along an arc with a radius of 5 mm.

At all boundaries, the tangential (*τ*) and normal (*σ_n_*) stresses were equal to zero (4), except for the one under load.

Flexural stresses and strains of the composites were determined by the following formulas:

(11) $$\sigma_{F}=\frac{3LF}{2b\;H^{2}},$$

(12) $$\varepsilon_{F}=\frac{6Hy_{na}}{L^{2}},$$

where *F* is the load on the sample; *b* is the sample width; *y_na_* is deflection of the neutral axis, *H* is the sample height; *L* is the support span.

**Figure 19:**
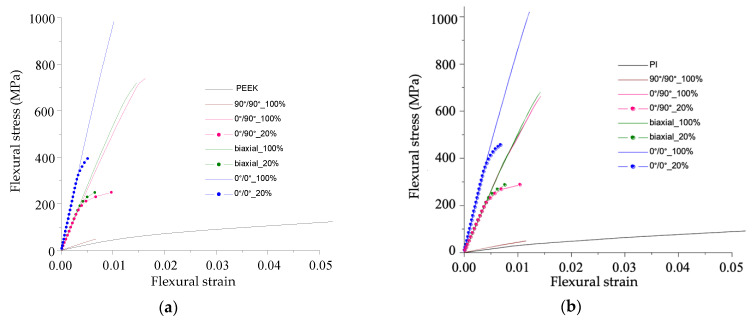
The flexural stress–strain curves for the PEEK- (**a**) and PI-based (**b**) composites with changing the CF layouts and adhesion levels (100% and 20%).

Force was calculated by summing the obtained stresses, multiplied by the load distribution area. Achievements of the material yield point (strength) by stresses in 10% of the sample volume was taken as a criterion for terminating the calculation process. The flexural stress–strain curves are shown in Figure 19 for the PEEK- (a) and PI-based (b) composites with changing the CF layouts and adhesion levels (100% corresponded to ultimate stresses equal to the yield point).

The obtained results differed slightly for the PEEK- and PI-based composites at the same adhesion level (Figure 18). At the same time, flexural strength was much higher than the experimental one for almost all composites, taking into account the fact that the adhesion level was 100%. An exception occurred for the ones with a transverse CF layout (90°/90°), the dependencies for which were close to the experimental curves (Figure 4a).

To compare the calculated results with the experimental data, the composites with a decrease in adhesion level of up to 20% were considered (Figure 19), in layers with the longitudinal CF layout. In this case, the obtained strength was closer to the experimental values (Figure 4, Figure 5 and Figure 6) than with the ideal adhesion. Due to the fact that the flexural test results did not take into account delamination and the number of contact points in an explicit form because of the simulation complexity, the slope of the obtained curves turned out to be greater than in the experiment. Thus, the calculated results showed (Figure 18) that the achieved adhesion level in the studied composites was about 20% (at the maximum level equal to the matrix yield point), and their flexural strength can be improved by several times through enhancing adhesion.

## 4. Conclusions

The laminated composites were fabricated with two types of high-strength thermoplastic binders reinforced with continuous unidirectional carbon fibers. Under the three-point bending conditions, they showed predominantly ductile fracture (a greater damage tolerance). The results of the studies of their structure, as well as theoretical and experimental investigations of the deformation behavior, enabled us to draw the following conclusions.

1. The laminated composites reinforced with continuous unidirectional CF oriented at (0°/0°) showed the maximum mechanical properties (both flexural modulus and strength) in the three-point flexural tests. The PEEK-based ones had twice the flexural strength compared to the PI-based samples (0.4 and 0.2 GPa, respectively), and the flexural modulus was four times higher (61 and 15 GPa, correspondingly). The difference in their flexural strength was not proportional to the variations of the respective characteristics of the used polymer binders.
2. It was shown that the interlayer boundary between the PEEK film and CF was straight and did not contain any discontinuities. In addition, the molten polymer had effectively impregnated the CF layer to form a wavy interface. However, this transition layer contained defects, primarily microcracks. At the same time, satisfactory interfacial adhesion between the binder and individual CF was ensured.
3. In the laminated PI-based composite, the straight interface without any discontinuities was observed between the polymer and CF tape layers. However, this polymer did not infiltrate the near-boundary zone of the CF tape to the required extent due to its lower MFI. As a result, pronounced delamination up to several tens of micrometers width was found inside the CF layer (tape) of the PI-based composite. There were no visible microcracks in this transition layer, and interfacial adhesion between the binder and the individual CF was satisfactory as well.
4. It was found that the use of the PI powder for manufacturing the laminated CF reinforced composites was not very promising due to their low mechanical properties (in comparison with the PEEK-based ones). The reason was that the lower MFI of the PI powder resulted in the presence of interlayer discontinuities.
5. A model of the CF reinforced composites was developed, taking into account discontinuities in the material, delamination between the polymer and CF, as well as the composite failure processes. On its basis, the effect of delamination and fracture on the strength properties was shown for both PEEK- and PI-based composites at various stress–strain states.
6. It was reported that increasing CF/polymer adhesion enhanced the tensile strength of longitudinally reinforced composites but did not change their elasticity modulus (corresponding quantitative estimates were obtained).
7. It was observed that the matrix type played a decisive influence on the properties of the composites reinforced in the transverse direction under tension. Increasing both adhesion and contact areas (number of contacting nodes) between CF and the polymers improved the strength characteristics of the composites. Similar results were obtained for the ones reinforced in the longitudinal direction under shear loads. The decrease in the number of contact points (simulating an increase in the number of defects) significantly reduced both shear modulus and strength.
8. The simulation results showed that the achieved adhesion level in the studied composites was about 20%, and their flexural strength can be improved by several times through enhancing adhesion. In theory, PEEK- and PI-based composites can have comparable properties at the same adhesion level assessed relative to the polymer matrix yield point.

## Figures and Tables

**Table 1:** MFI of polymers.

	Sample Type	MFI, g/10 min
1	PEEK powder	7.92 ± 0.44
2	PEEK film/CF	8.25 ± 0.23
3	PI powder	4.56 ± 0.53

**Table 2:** The physical and mechanical properties of the CF reinforced composites.

	Sample	$\mathrm{Flexural}\;\mathrm{Modulus}\;G_{B}^{U},\;\mathrm{GPa}$	$\mathrm{Flexural}\;\mathrm{Strength}\;\;\sigma_{B}^{U},\;\mathrm{MPa}$	$\mathrm{Fracture}\;\mathrm{Strain}\;{\varepsilon_{B}^{U}}_{\;}$
1	PEEK powder/CF (0°/0°)	55.4 ± 2.1	443 ± 24	0.020 ± 0.005
2	PEEK film/CF (0°/0°)	59.5 ± 3.7	422.8 ± 12.5	0.034 ± 0.001

**Table 3:** The physical and mechanical properties of the CF reinforced composites and neat polymers.

	Sample	Density, g/cm^3^	$\mathrm{Flexural}\;\mathrm{Modulus}\;G_{B}^{U},\;\mathrm{GPa}$	$\mathrm{Flexural}\;\mathrm{Strength}\;\sigma_{B}^{U},\;\mathrm{MPa}$	$\mathrm{Fracture}\;\mathrm{Strain}\;{\varepsilon_{B}^{U}}_{\;}$
1	PEEK	1.30	4.2 ± 0.6	166.2 ± 6.1	0.050 ± 0.005
2	PEEK/CF (0°/0°)	1.54	59.5 ± 3.7	422.8 ± 12.5	0.034 ± 0.001
3	PEEK/CF (90°/90°)	1.51	4.1 ± 0.3	42.3 ± 6.4	0.007 ± 0.004
4	PEEK/CF (0°/90°)	1.46	19.8 ± 7.2	173.8 ± 26.8	0.022 ± 0.001
5	PEEK/CF (biaxial)	1.33	27.4 ± 0.3	277.2 ± 34.9	0.014 ± 0.001
6	PI	1.37	2.4 ± 0.4	143.3 ± 6.2	0.050 ± 0.001
7	PI/CF (0°/0°)	1.40	15.0 ± 2.0	190.2 ± 18.7	0.035 ± 0.001
8	PI/CF (90°/90°)	1.43	4.1 ± 0.5	30.1 ± 4.1	0.010 ± 0.001
9	PI/CF (0°/90°)	1.33	3.8 ± 1.1	43.0 ± 5.2	0.050 ± 0.001
10	PI/CF (biaxial)	1.38	10.8 ± 1.1	158.4 ± 1.2	0.040 ± 0.001

## Data Availability

The data presented in this study are available on request from the corresponding author.

## Prospects

According to the above conclusions, the further research direction is associated with the development of technological aspects of the formation of the laminated composites, minimizing the number of discontinuities, as well as increasing interfacial and interlayer adhesion. In particular, CF surfaces will be activated by electric-discharge plasma treatment.

## Nomenclature

PEEK	Polyetheretherketone
PI	Polyimide
CF	Carbon fiber
MFI	Melt flow index
FEM	Finite element method
$G_{B}^{U}$	Flexural modulus
$\sigma_{B}^{U}$	Bending strength
$\varepsilon_{B}^{U}$	Flextural stain
*σ_F_*	Flexural stress
*ε* *_F_*	Flexural strain
*τ*	Tangential stress
$\sigma_{n}$	Normal stress
$\sigma_{YS}$	Yield point
$\sigma_{a}$	Maximum adhesion level
$\sigma_{x}$	Longitudinal composite tensile strength (along *x*-axis)
$\sigma_{y}$	Transverse composite tensile strength (along *y*-axis)
$E_{f}$	Fiber elastic modulus
$E_{m}$	Matrix elastic modulus
$G_{f}$	Fiber shear modulus
$G_{m}$	Matrix shear modulus
$G_{x}$	Composite shear modulus along the *x*-axis
$E_{x},E_{y}$	Composite elastic modulus along the *x* and *y* axis
*ϕ*	CF volumetric content
*v*, *u*	Total displacement along the *y* and *x* axis
*v_i_*, *u_i_*	Displacement along the *y* and *x* axis for one step
*k*, *m*, *l*	Node numbers
*F*	Load on sample
*b*	Sample width
*y_na_*	Deflection of the neutral axis
*H*	Sample height
*L*	Support span
